# Editorial: Environmental omics and their biotechnological applications

**DOI:** 10.3389/fmicb.2023.1165558

**Published:** 2023-03-09

**Authors:** Rosa María Martínez-Espinosa, Jean Armengaud, Sabine Matallana-Surget, Alfonso Olaya-Abril

**Affiliations:** ^1^Multidisciplinary Institute for Environmental Studies “Ramón Margalef”, University of Alicante, Alicante, Spain; ^2^Département Médicaments et Technologies pour la Santé (DMTS), Université Paris-Saclay, CEA, INRAE, Bagnols-sur-Cèze, France; ^3^Division of Biological and Environmental Sciences (BES), Faculty of Natural Sciences, University of Stirling, Stirling, United Kingdom; ^4^Department of Biochemistry and Molecular Biology, University of Córdoba, Córdoba, Spain

**Keywords:** environmental omics approaches, genomics, transcriptomics, proteomics, biotechnological applications

The refinement of omics methods over recent years has enabled significant advances in microbiology. The functioning of the environment is extremely diverse, complex, and challenging to decipher, and consequently requires advanced molecular tools to address key scientific questions that remain poorly documented. Environmental omics aim at a better understanding of the metabolic processes of a wide range of organisms and/or complex microbial communities to improve phenotype-genotype linkages, thus providing novel insights into the key molecular players in response to environmental changes and invaluable information on microbial communities. In this context, this Research Topic showcases the power of environmental omics to characterize novel catalysts for biotechnological applications. The range of applications is large and includes the bioremediation of pollutants, design of innovative biosensors, screening for novel catalysts and therapeutic drugs, and bioproduction of novel chemicals and materials. We have encouraged scientists using omics to study different aspects of environmental processes to contribute to this Research Topic. The knowledge generated with these studies could be complemented by others with the ultimate goal of properly characterizing processes to develop knowledge-based biotechnological applications.

The optimization of metabolic processes for improving the performance of a bioprocess requires gaining in-depth knowledge, not only to define the elements directly involved but also those necessary to supply all the components and balance metabolic flows. Omics facilitate the generation of knowledge, providing holistic and global information on the processes under examination. Likewise, its development has enabled the progress of studies beyond the simple molecular study of axenic cultures or single model organisms to more complex experimental designs using microbial consortia (either natural or artificial). In this way, previously unknown functions could be discovered and annotated. However, improving methodologies and reducing their costs are of utmost importance for their widespread use. In this regard, Abdelmoneim et al. proposed in this Research Topic to replace the classical 16S RNA gene amplicon analysis with a taxon-specific qPCR for increasing the throughput of soil surveys which could also be used as a diagnostic tool for soil health. This study is an excellent illustration of how omics will become valuable diagnostic tools over the next decade, as recently envisioned (Armengaud, 2023).

The potential biotechnological applications of environmental processes are incredibly varied. To illustrate this, we could highlight how the discovery of comammox in the chemolithoautotrophic *Nitrospira* has enabled the development of nitrogen removal strategies in wastewater treatment plants (Mehrani et al., 2020) and how research continues to optimize this process (Lawson et al., 2021). Nitrogen fixation by plants is another major Research Topic being developed by various research groups, such as that of Luis Rubio and colleagues (He et al., 2022). However, other factors, such as the availability of phosphorus, affect biological nitrogen fixation, and could be important bottlenecks. In the case of phosphorus, the insertion of the non-specific alkaline phosphatase PhoX could be considered to improve phosphorus intake. In fact, PhoX is primarily responsible for the phosphorus solubilization activity in proteobacteria, even when nitrogen fixation and phosphorous solubilization occur simultaneously in *Azotobacter chroococcum* NCIMB 8003. This was demonstrated by Biełło et al., who also revealed how domain enrichment analysis is particularly suitable as the approach goes beyond the classical enrichments in gene ontology terms and metabolic pathways (Geller et al., 2021; Verstraeten et al., 2021). It is noteworthy that the PhoX enzyme was misannotated, but its function could be properly assigned in this study. We are confident that such methodology could be successfully applied to environmental samples or enriched samples with certain types of organisms and analyzed by meta-omics.

Global warming endangers agriculture yields and urgent solutions should be proposed (Armengaud, 2022). Environmental omics may be key to increasing the yield of crop plants by minimizing the environmental risks, developing biofertilizers (Irineu et al., 2022), or producing biostimulants (Ahmad et al., 2022). Biodegradation and bioremediation are also important topics for restoring the environment in which omics are being increasingly applied. In this Research Topic, Llorca and Martínez-Espinosa published a straightforward application of omics with a thorough analysis of the bioremediation potential against copper of the haloarchaeon *Haloferax mediterranei* ATCC 33500. Another study devoted to the resilience of microorganisms to natural toxicants is presented by Grosjean et al.. In this original work, *Saccharomyces cerevisiae* mutants and their screening with high-throughput proteomics made it possible to identify the importance of the cell wall as a shield to discriminate light and heavy lanthanides. This allows for a better understanding of eukaryotes.

Once a process has been extensively characterized using omics, it could be copied and integrated with the systems biology approach (SynBio) into an efficient industrial process. Approaches such as design-build-test-learn cycles are well suited for this purpose (Dvorák et al., 2017). Whatever the purpose, from the generation of fully synthetic pathways, such as the *in vitro* CO_2_ fixation (Schwander et al., 2016), to the optimization of natural processes, a critical decision should be made: whether to use cell-dependent or cell-free systems. This will be especially relevant when, during the process under study, toxic compounds are produced, as in the classic example of protoanemonin in the degradation of PCBs (Blasco et al., 1997) or the one described by Chen et al. in this Research Topic. In this work, the toxic effect of dimethyl phthalate, a widely used plasticizer, on the *Pseudomonas fluorescens* bacterium was reported and its molecular consequences were described in great detail. These results could restrict the possibility of using this bacterium as a plastic degrader. The two options, to use the catalysis in a cell-free system or to increase the tolerance of the *P. fluorescens* chassis, could be winning alternatives.

The works presented in this Research Topic illustrate different facets of the great utility of omics approaches for the rational development of a large variety of biotechnological applications. That the continuous progress in these methodologies are becoming more affordable will allow us to explore and understand new environments and processes to develop and optimize novel biotechnological applications.

## Author contributions

AO-A conceptualized and drafted the editorial. RM-E, JA, and SM-S provided critical review and insight to improve the writing. All authors approved the final paper.
